# Supplementary material for: Novel Hydroxyl-Functional Aliphatic CO2-Based Polycarbonates: Synthesis and Properties
Source: Int J Mol Sci. 2025 Oct 18;26(20):10151. doi: 10.3390/ijms262010151 (PMC12563446; doi:10.3390/ijms262010151)
Supplement: Supplementary file 1 [file ijms-26-10151-s001.zip › ijms-3923610-supplementary.pdf]

Supplementary Information

Novel hydroxyl-functional aliphatic CO_2-based polycarbonates: synthesis and properties

Nikita M. Maximov,[a] Sergey A. Rzhevskiy,[b] Andrey F. Asachenko,[*a,b] Anna V. Plutalova,[a] Elena S. Trofimchuk,[a] Evgenii A. Lysenko,[a,c] Olga V.Shurupova,[b] Ekaterina S. Tarasova,[b] Elena V. Chernikova,[*a,b] and Irina P. Beletskaya[a,b]

[1] Lomonosov Moscow State University, Faculty of Chemistry, Russian Federation, 119991, Moscow, Lenin Hills, 1, bld. 3;nmm33@mail.ru (N. M. N), annaplutalova@gmail.com (A. V. P), elena_trofimchuk@mail.ru (E.S.T.), chernikova_elena@mail.ru (E.V.C.); beletska@org.chem.msu.ru (I. P. B)

[2] A.V. Topchiev Institute of Petrochemical Synthesis of Russian Academy of Sciences, Russian Federation, 119991, Moscow, Leninsky Av.; rs89a@yandex.ru(S.A.R.); aasachenko@ips.ac.ru (A. F. A), shurupovao@yandex.ru (O. V. S.), ekatya99@bk.ru (E. S. T.)

[3] Shenzhen MSU-BIT University, Faculty of Materials Science, China, 518172, No. 1 International University Park Road, Longgang District, Shenzhen; evglys1970@gmail.com (E. A. L)

* Correspondence: aasachenko@ips.ac.ru (A.F.A); chernikova_elena@mail.ru (E.V.C)

Table S1. Compositions of reaction mixtures taken for the terpolymerization

m_{Cat}, mg	$m_{co\text{-}Cat}$, mg	PO		SolGE		f_{Sol}
		mg	mmol	mg	mmol	
5.2	4.3	1666	28.7	564	3.0	0.10
10.0	7.6	3010	51.8	2376	12.6	0.21
9.8	7.7	2617	45.0	3607	19.2	0.32
10.5	8.0	2254	38.8	4785	25.4	0.43
10.5	8.3	1845	31.8	5980	31.8	0.53
10.0	8.0	1533	26.4	7143	38.0	0,61
10.0	8.2	1162	20.0	8385	44.6	0.70
10.0	7.3	738	12.7	9572	50.9	0.81
10.0	7.3	369	6.3	10769	57.3	0.91

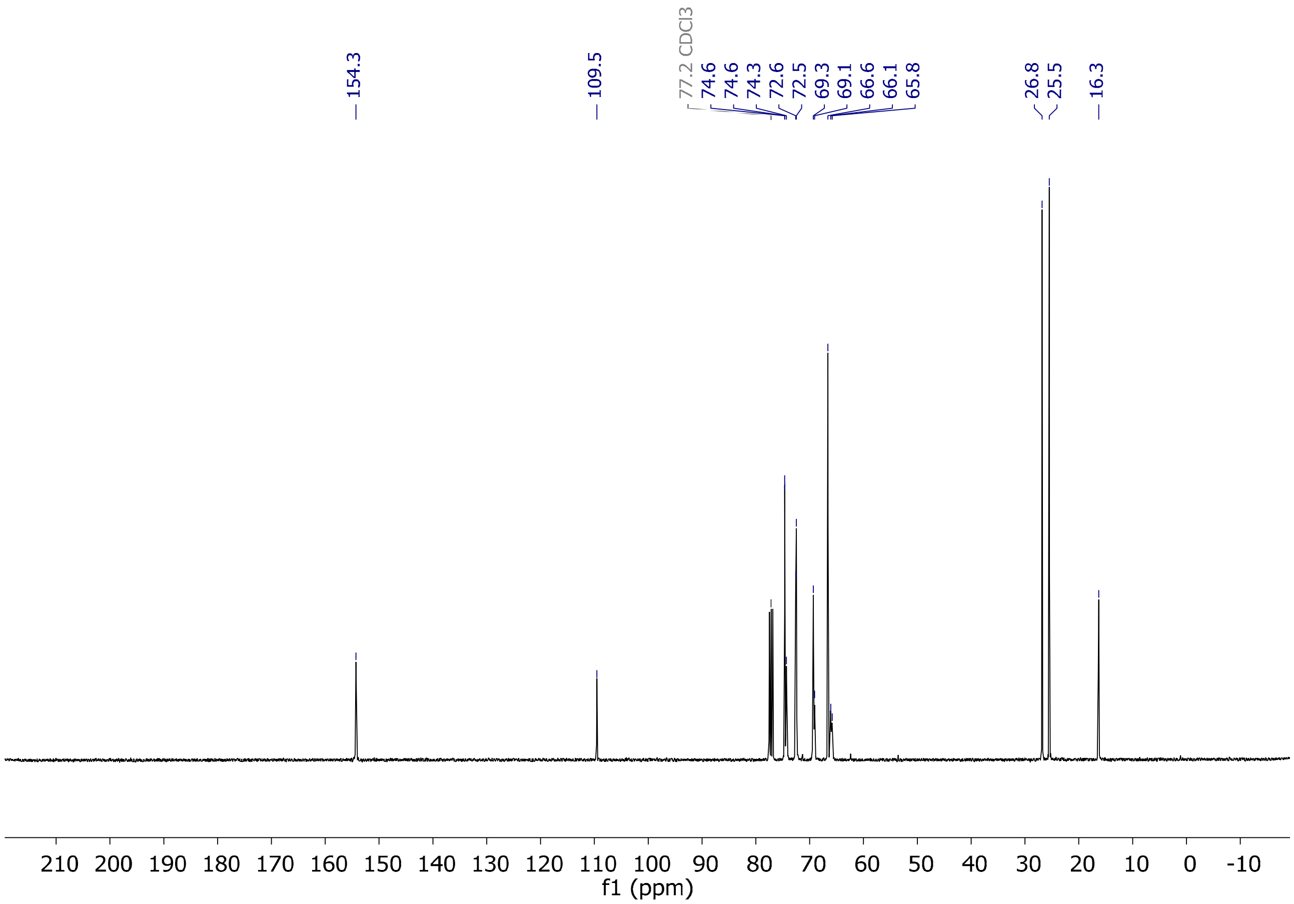

Figure S1. ^{13}C NMR (b) spectra of the synthesized poly(solketal glycidyl ether carbonate-co-propylene carbonate) from the monomer feed containing f_{SolGE} = 0.53.

Calculation of the monomer conversion and selectivity

To determine the conversion and selectivity of the polymerization process from the NMR data of the reaction mixture, Eqs. S1 – S5 are used. Both protons of cyclic carbonates (H-7, H-7′) can be detected superimposed in the spectrum at 4.83 ppm, while one of the protons from PC (H-6) is identified as a separate triplet at 4.53 ppm.

$$f_{\text{SolGE}} = \frac{Ib}{Ib+Ig} \qquad \text{(S1)}$$

$$Conv.SolGE = \left(\frac{I_1-5I_2}{I_1}\right) * 100\% \quad \text{or} \quad Conv.SolGE = \frac{I_3}{I_3+I_4} * 100\% \qquad \text{(S2)}$$

$$Conv.PO = 5f_{\text{SolGE}}\frac{I_5+I_6-0.002*I_1*Conv.SolGE}{I_1*(1-f_{\text{SolGE}})} \quad \text{(S3)}$$

$$Sel.PO = \frac{I_5-0.002*I_1*Conv.SolGE}{\mathrm{I}_5+\mathrm{I}_6-0.002*I_1*Conv.SolGE} \qquad \text{(S4)}$$

$$Sel.SolGE = \frac{0.002*I_1*Conv.SolGE}{0.002*I_1*Conv.SolGE-I_7+I_6} \qquad \text{(S5)}$$

The typical spectra of the monomer mixture, the reacted mixture after polymerization and the purified polycarbonate are shown in Figure S2. We assume that SolGE residues remain in the autoclave after opening following polymerization, unlike unreacted propylene oxide, which is evacuated. Figure S3 provides an example of calculating integral intensities to determine conversion and selectivity.

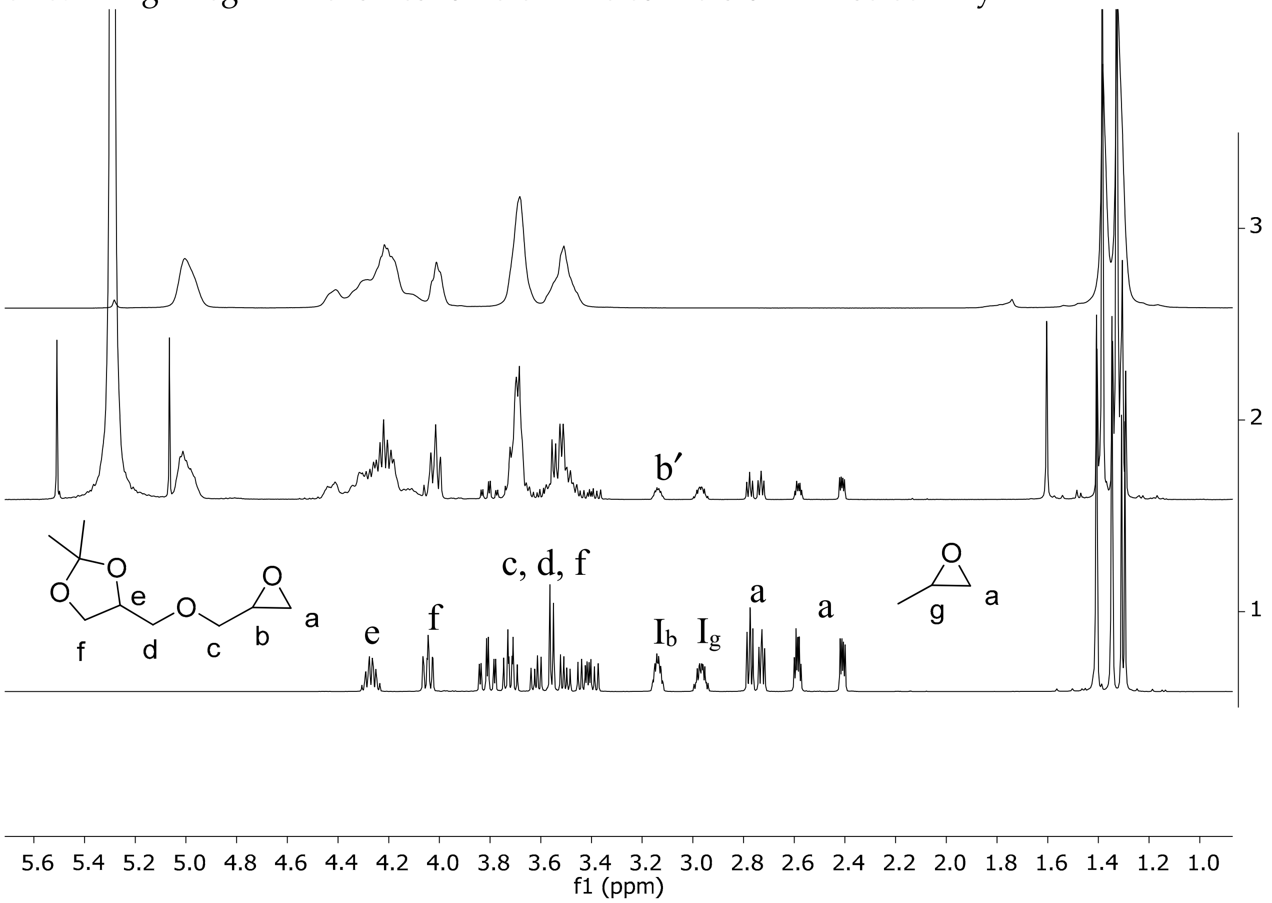

Figure S2. ^{1}H NMR spectra of the synthesized poly(glyceryl glycerol carbonate-*co*-propylene carbonate) from the monomer feed containing f_{SolGE} = 0.53 (3), reaction mixture at 24 h (2), monomer pre-mixture (1).

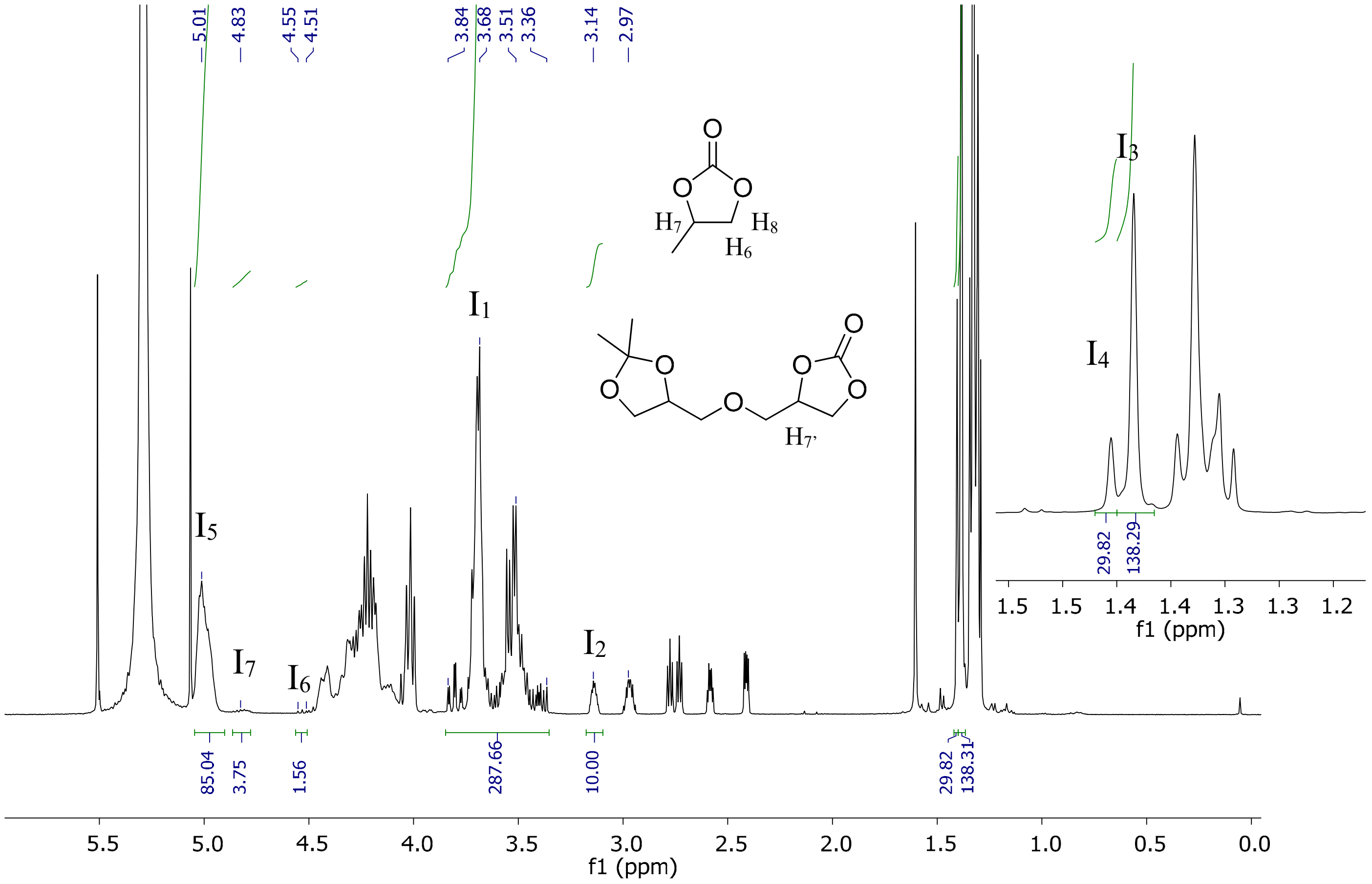

Figure S3. Example of the analysis of ^{1}H NMR spectrum of reaction mixture (f_{SolGE} = 0.53)

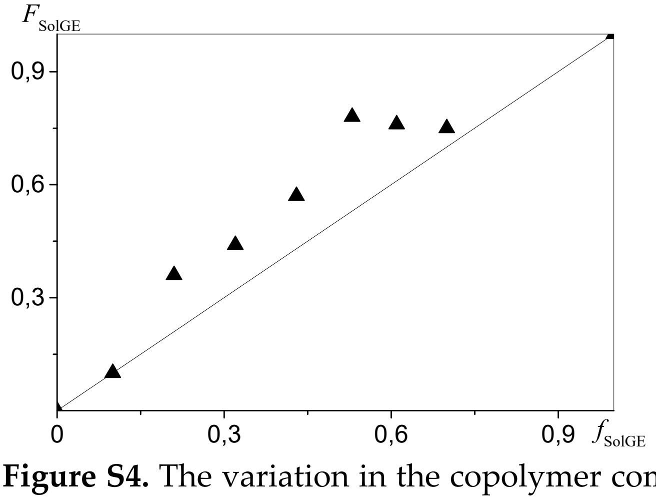

Figure S4. The variation in the copolymer composition as a function of the monomer feed for polycarbonates formed during terpolymerization of SolGE, PO, and CO_2.

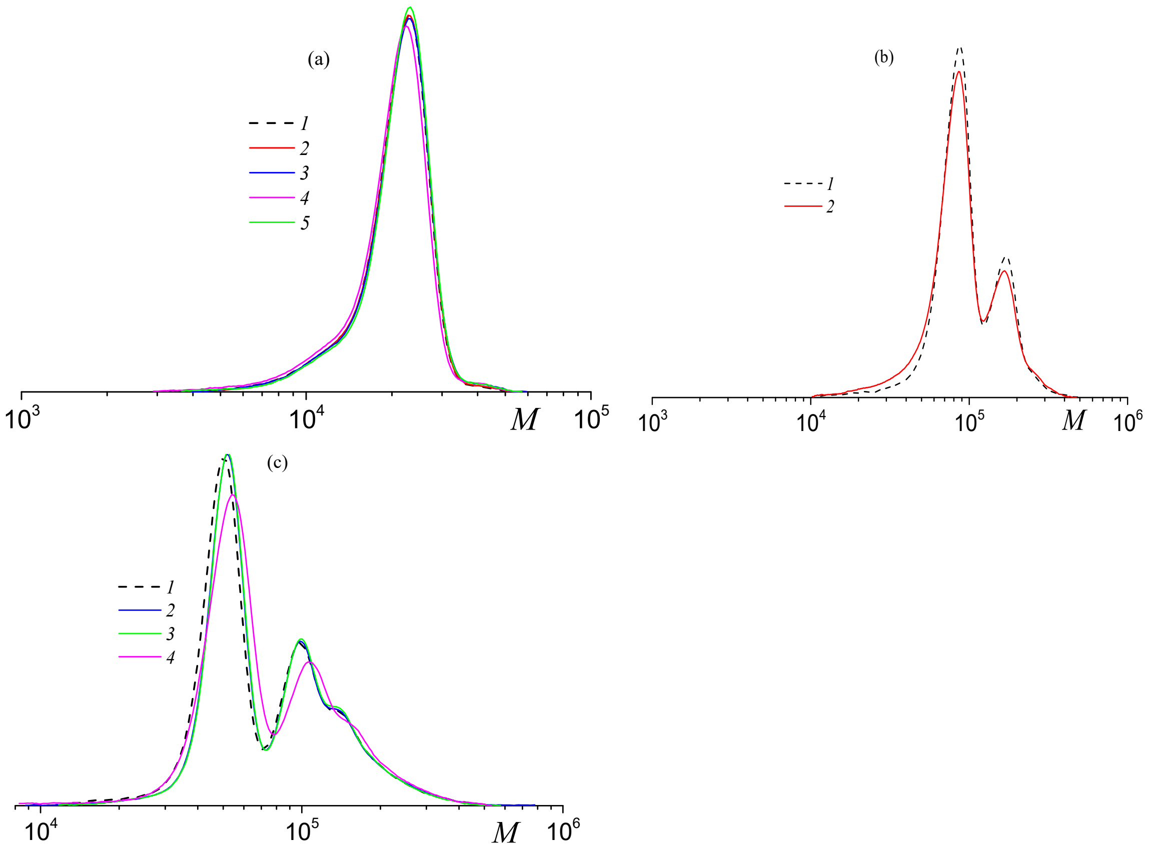

Figure S5. MWDs of poly(solketal glycidyl ether carbonate-*co*-propylene carbonate) before (1) and after deprotection (2 – 5). (a) Samples 3 (2), 4 (3), 5 (4), and 6 (5); (b) sample 2 (2); (c) samples 9 (2), 10 (3), and 11 (4). Numbering of the samples in the Figure corresponds to their numbering in Table 5.

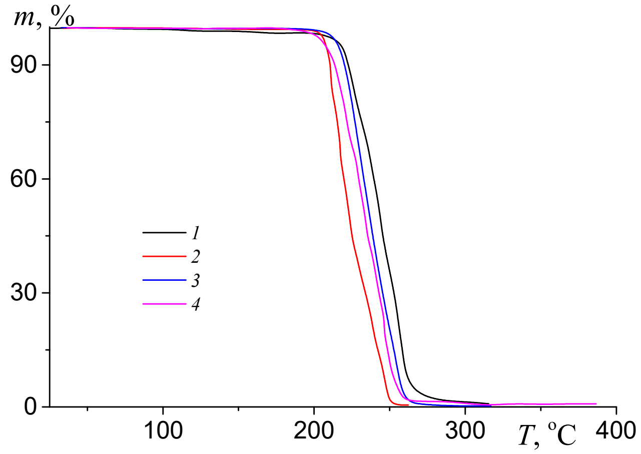

Figure S6. TGA curves recorded in air for terpolymers with f_{SolGE} = 0.11 (1), 0.22 (2), 0.32 (3), and 0.53 (4).

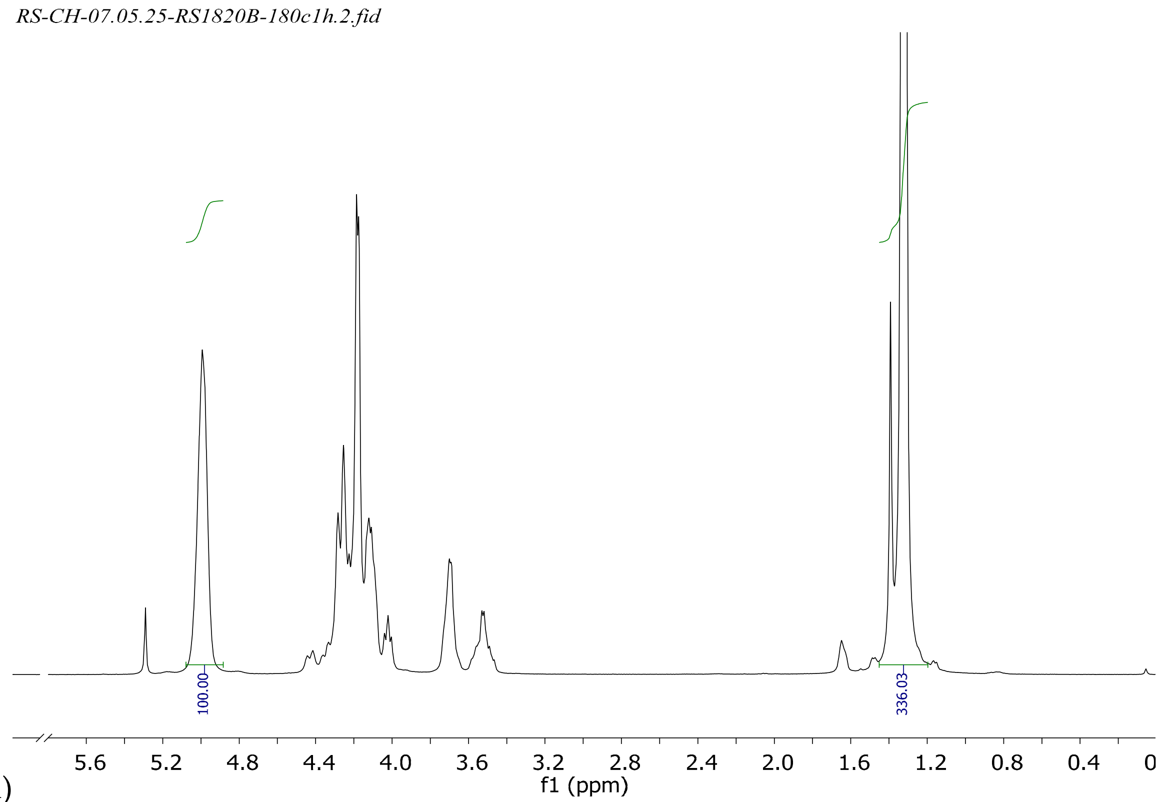
RS-CH-07.05.25-RS1820B-180c1h.2.fid
100.00
336.03
5.6 5.2 4.8 4.4 4.0 3.6 3.2 2.8 2.4 2.0 1.6 1.2 0.8 0.4 0
f1 (ppm)

(a)

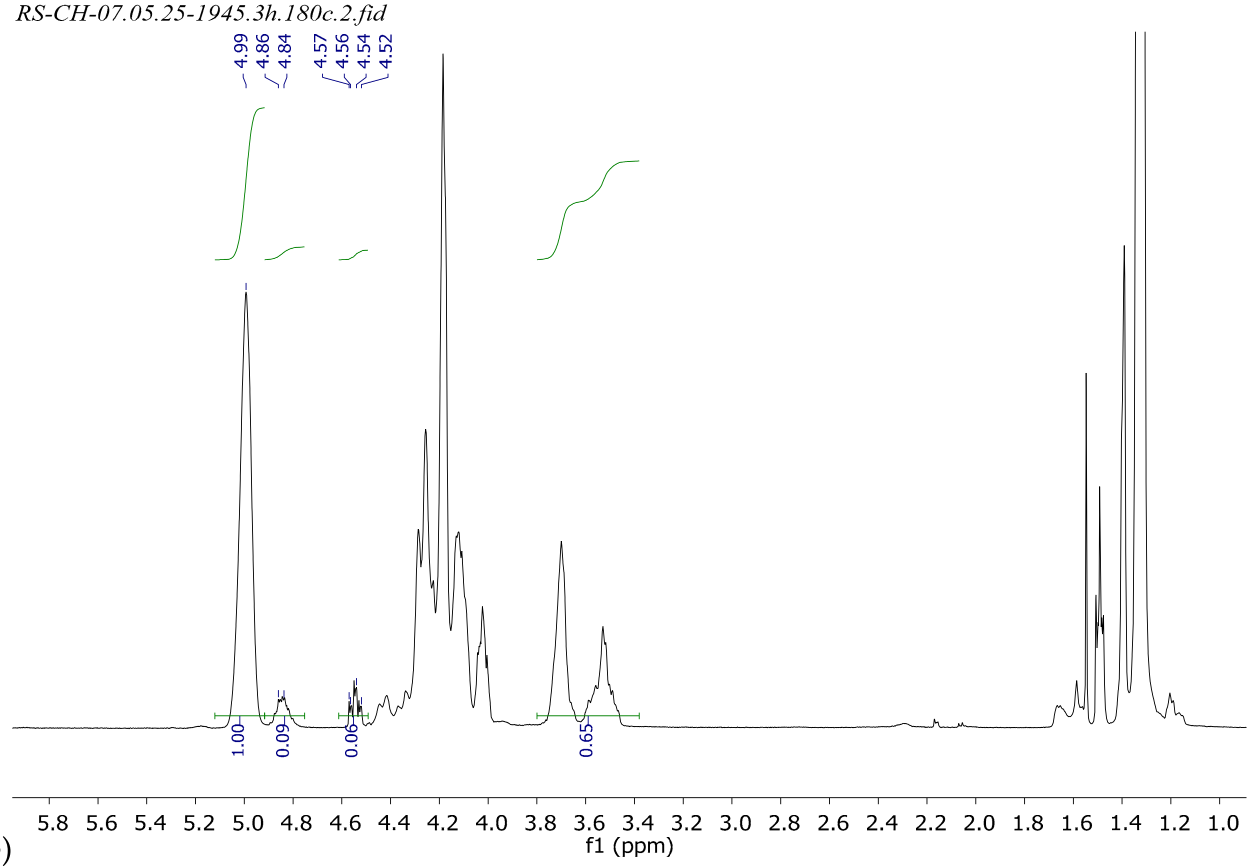
RS-CH-07.05.25-1945.3h.180c.2.fid
4.99
4.86
4.84
4.57
4.56
4.54
4.52
1.00
0.09
0.06
0.65
5.8 5.6 5.4 5.2 5.0 4.8 4.6 4.4 4.2 4.0 3.8 3.6 3.4 3.2 3.0 2.8 2.6 2.4 2.2 2.0 1.8 1.6 1.4 1.2 1.0
f1 (ppm)

(b)

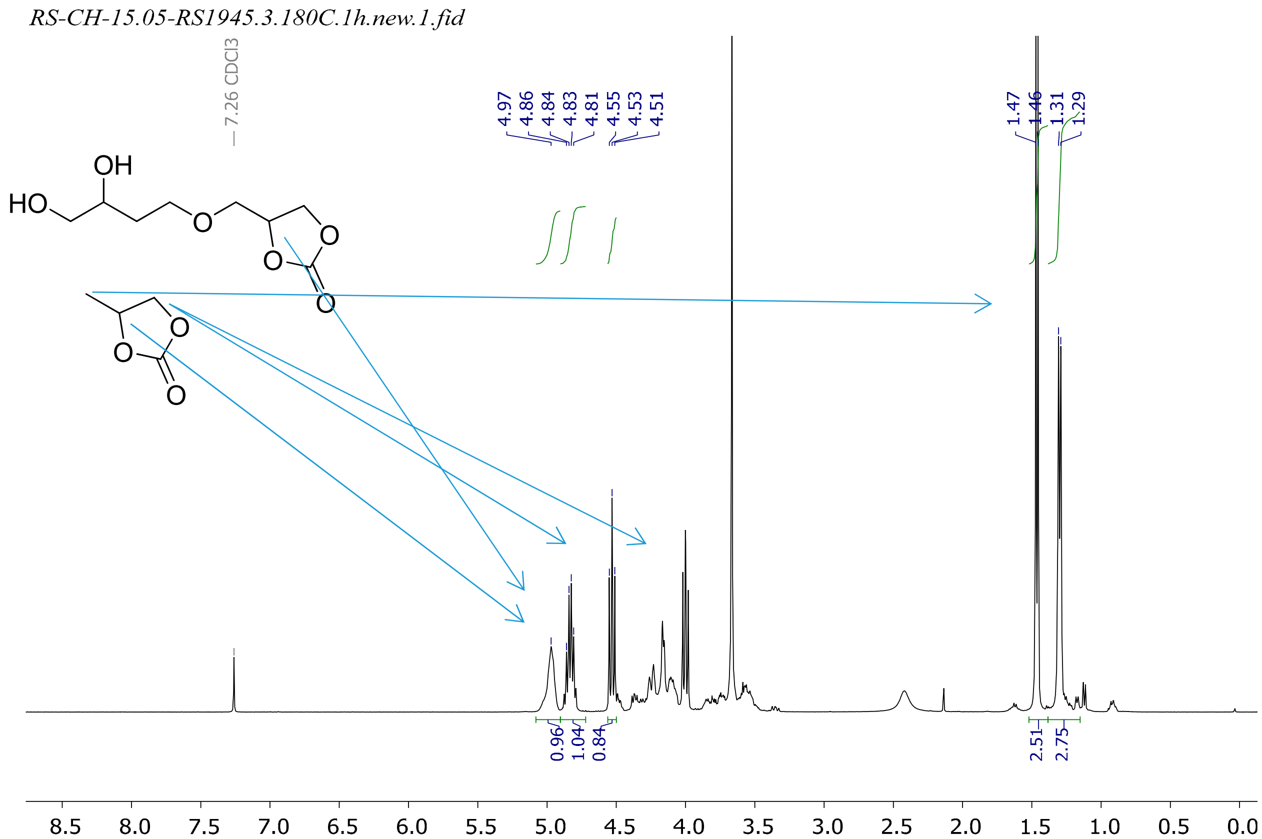

(c)

Figure S7. ^{1}H NMR spectra of the products of the thermal treatment of polycarbonates (f_{SolGE} = 0.11) at 180°C in vacuum during 1 (a, c) and 3h (b). (a) Protected, $M_{n1} = 85.3\times10^3$, $M_{n2} = 130.3\times10^3$; (b) protected, $M_n = 19.4\times10^3$; (c) deprotected, $M_n = 6.3\times10^3$.
